# Biopolymeric nanoparticles based effective delivery of bioactive compounds toward the sustainable development of anticancerous therapeutics

**DOI:** 10.3389/fnut.2022.963413

**Published:** 2022-07-15

**Authors:** Neelam Pathak, Pankaj Singh, Pradeep Kumar Singh, Swati Sharma, Rajat Pratap Singh, Anmol Gupta, Richa Mishra, Vivek Kumar Mishra, Manikant Tripathi

**Affiliations:** ^1^Department of Biochemistry, Dr. Rammanohar Lohia Avadh University, Ayodhya, India; ^2^Biotechnology Programme, Dr. Rammanohar Lohia Avadh University, Ayodhya, India; ^3^Department of Biosciences, Integral University, Lucknow, India; ^4^Department of Biotechnology, Guru Ghasidas Vishwavidyalaya, Bilaspur, India; ^5^Department of Microbiology, King George Medical University, Lucknow, India

**Keywords:** biopolymeric nanoparticles, cancer treatment, drug delivery, nanomedicines, natural bioactives

## Abstract

Nowadays, effective cancer therapy is a global concern, and recent advances in nanomedicine are crucial. Cancer is one of the major fatal diseases and a leading cause of death globally. Nanotechnology provides rapidly evolving delivery systems in science for treating diseases in a site-specific manner using natural bioactive compounds, which are gaining widespread attention. Nanotechnology combined with bioactives is a very appealing and relatively new area in cancer treatment. Natural bioactive compounds have the potential to be employed as a chemotherapeutic agent in the treatment of cancer, in addition to their nutritional benefits. Alginate, pullulan, cellulose, polylactic acid, chitosan, and other biopolymers have been effectively used in the delivery of therapeutics to a specific site. Because of their biodegradability, biopolymeric nanoparticles (BNPs) have received a lot of attention in the development of new anticancer drug delivery systems. Biopolymer-based nanoparticle systems can be made in a variety of ways. These systems have developed as a cost-effective and environmentally friendly solution to boost treatment efficacy. Effective drug delivery systems with improved availability, increased selectivity, and lower toxicity are needed. Recent research findings and current knowledge on the use of BNPs in the administration of bioactive chemicals in cancer therapy are summarized in this review.

## Introduction

Non–communicable diseases, such as cancer, are one of the leading causes of death worldwide. The most common type of cancer is lung cancer, which is followed by breast, prostate, and colon cancer (1). Within the next two decades, the number of new cases is expected to increase by almost 70% (2). Present treatment options for cancer include surgery, chemotherapy, and/or radiation therapy. But there are some challenges associated with the above treatment strategies like radiation therapy or chemotherapeutic drugs not only damage cancerous cells, but also harm healthy cells (3). The degree of side effects may vary from person to person depending on the type of cancer, and other variables. Chemotherapy hinders the DNA synthesis in fast–dividing cancer cells, leading to their death. It leads to various side effects as it affects healthy cells of the body (4). Additionally, high drug doses used during chemotherapy lead to increased toxicity to normal cells, with the risk of development of drug resistance (4). Thus, a major task is to strengthen the selectivity of anticancer drugs against cancerous cells while sparing normal cells and tissues. In order to treat cancer, efficient targeted delivery methods of chemotherapeutic drugs or anticancer bioactive molecules are also required. In view of the above facts, a potential approach was used to target tumor cells through nano–medicine–based preparations (5). These preparations consist of ultra–micrometer–sized carriers having the bioactive compound(s) that are capable of selectively treating tumors, thus, improving the therapeutic value of the anticancer drugs that are being used.

For the past decade, nanoparticles (NPs) have become tremendously appealing for use in biomedicine. Through enhanced permeability and retention (EPR) effects in tumor tissues, nanoparticle-based drug delivery techniques or nanocarriers can improve therapeutic efficacy and selectivity (6). Nanocarriers also have superior cellular uptake as compared to standard chemotherapeutic molecules. Among the nanocarriers, polymeric NPs, micelles, and liposomes have received significant attention (7).

The polymers utilized to prepare polymeric NPs such as poly (methyl methacrylate), polystyrene, polyacrylates, and polyacrylamide were not eco–friendly (8). The nanosystems prepared by these materials displayed chronic toxicity and inflammatory reactions. The use of new nanosystems might have huge interest to accomplish additional applications for the current large–scale characterized bioactive compounds. Nanoencapsulation, in actuality, is a low-cost technology that could allow for regulated bioactives release, which could be useful in cancer treatment. There are synthetic and natural biodegradable polymers. Polymeric nanoparticles have been created using naturally occurring biodegradable polymers, such as alginate, gelatin, chitosan, and others (9). Some researchers studied about targeted delivery systems of therapeutics through chitosan and other biopolymer based NPs for cancer therapy (10, 11). Therefore, this review focuses on recent progresses in the development of BNPs for the effective delivery of bioactive chemicals for targeted therapy of cancer.

## Bioactive compounds

Bioactive substances are a natural occurrence as well as a part of our diet. Plants produce phytochemicals, which are natural bioactives capable of regulating metabolic processes and promoting good health (12). Amino acids, vitamins, enzymes, fibers, minerals, essential oils, and polyphenols are among the plant-based bioactive compounds (13). Natural biopolymers also consist of plant–derived or animal polysaccharides and proteins as well as polymers of microbial (bacteria, fungi, and yeast) sources. Several bioactive substances have the potential to be used as cancer chemotherapeutic drugs in past few years. Phytochemicals like vinca alkaloids, cephalotaxus, berberine, combretastatins, colchicine, ellipticine, and others are known for anticancerous activities against many cancer types (14). A flavonoid, chicoric acid isolated from *Echinacea purpurea* act as an immune stimulant by increasing the number of natural killer cells (15). A sulfur–containing compound called Ajoene isolated from *Allium sativum* suppresses the rate of cancer development. In a study, Shang et al. (16) reported the role of ajoene in the suppression of the growth of human breast cancer cells. Likewise, curcumin, an active chemical derived from the *Curcuma longa* plant, has anticancer properties. It caused the transcription factors AP1, NFkB, and STAT3 to be inhibited, halting the cell cycle in the G2/M phase (17). Similarly, another anticancer agent like Lappaol F has been obtained from *Arctium lappa* L. and has arrested the G2 phase of the cell cycle by promoting the G1 phase in which the p21 gene plays an important role for the same Lappaol F (18).

Citral is a bioactive ingredient found in citrus–based plant *Cymbopogon citratus*. Nanostructured lipid carrier–citral showed effective results against MDA–MB−231 breast cancer cells (19). Several researchers reported promising bioactive components from *Cannabis sativa* like delta 9-tetrahydrocannabinol (THC) and cannabinoids (CBD) which have also been investigated for their potential to treat cancer in both *in vivo* and *in vitro* studies (20–22).

A compound wedelolactone, isolated from *Eclipta alba* involve in the inhibition of T47D, MCF−7, and MDAMB−231 cells by exciting ER signaling (23). Curcumin is another important bioactive obtained from *Curcuma longa* which contains curcuminoids compounds (24). Curcumin is known to have low bioavailability because of its poor absorption and fast removal from the body. Basniwal et al. (25) have used NPs mediated delivery of curcumin to increase its bioavailability. Curcumin has been reported as a potent anticancer agent on various types of human cancers such as lung, pancreatic, prostate, breast, ovarian and others (26). Another bioactive myricetin is a bioflavonoid commonly present in food sources such as berries, red wine, vegetables, tea, and medicinal plants which is a promising anti–carcinogen with therapeutic potential reported in the breast (27), colon (28), liver (29), ovarian (30), and skin cancers (31). Geraniin is a dehydroellagitannin that is found in a variety of medicinal plants and is thought to be the most active natural chemical. Geraniin was discovered from the fruit of the *Phyllanthus emblica* L. plant, and found to have potent antioxidant, antimicrobial, and anticancer properties (32, 33). On MCF7 human breast cancer cells, geraniin was found to have an anticancer impact (34).

The two major classes of fat soluble antioxidants in Vitamin E are tocopherols and tocotrienols (T3) (35) that showed anticancerous properties against skin (36), stomach (37), liver (38), breast (39), colon (40), lung (41), and prostate cancer (42). Anticancer activity has been documented for a number of biologically active substances (Table 1).

**Table 1:** Biologically active molecules showing anticancerous activity.

**S. No**.	**Bioactive compounds/ molecules**	**Sources**	**Active molecules**	**Effects**	**References**
1.	Polyphenolic compounds	Red wine, chocolate, flaxseed oil, various plants	Corilagin	Carcinogen detoxification, inhibits tumor initiation/promotion, antimutagen	(43)
2.	Cyclopeptide	*Nostoc*	Cryptophycin	Anticancerous	(44)
3.	Ajoene	*Allium* sativm	Ajoene	Anticancerous	(45)
4.	Natural metabolites	*Marine Cyanobacteria*	Apratoxin A	Anticanceros	(46)
5.	Gallic acid	*Toona sinensis leaf extract*	–	ROS-mediated anticancerous activity against prostate cancer cells	(47)
6.	Polyphenol and flavonoid	Chlorella vulgaris	–	Inhibit lung cancer metastasis	(48)
7.	Microcystins	Cyanobacteria	Cryptophycins	Anticancer	(49)
8.	Vinorelbine, (Navelbine)	*Catharanthus roseus*	Vincristine	Anticancer activities lung cancer	(50)
9.	Geraniin	–	–	Anticancer activities	(32)
10.	Epigallocatechin-3-gallate	–	–	Prostate cancer prevention	(51)
11.	Flavonoids	*Artemisia annua*	Casticin	Anticancer	(52)
12.	Apigenin	–	–	Anticancerous	(53)
13.	Flavonoid	–	Luteolin	Anticancerous	(54)
14.	Terpene	–	Curcumin	Anticancerous	(55)
15.	Polyphenols	Derived from grapes, berries, red wine, peanuts	Resveratrol	Anticancerous	(56)

## Development of biopolymeric nanomaterials

**Figure 1:**
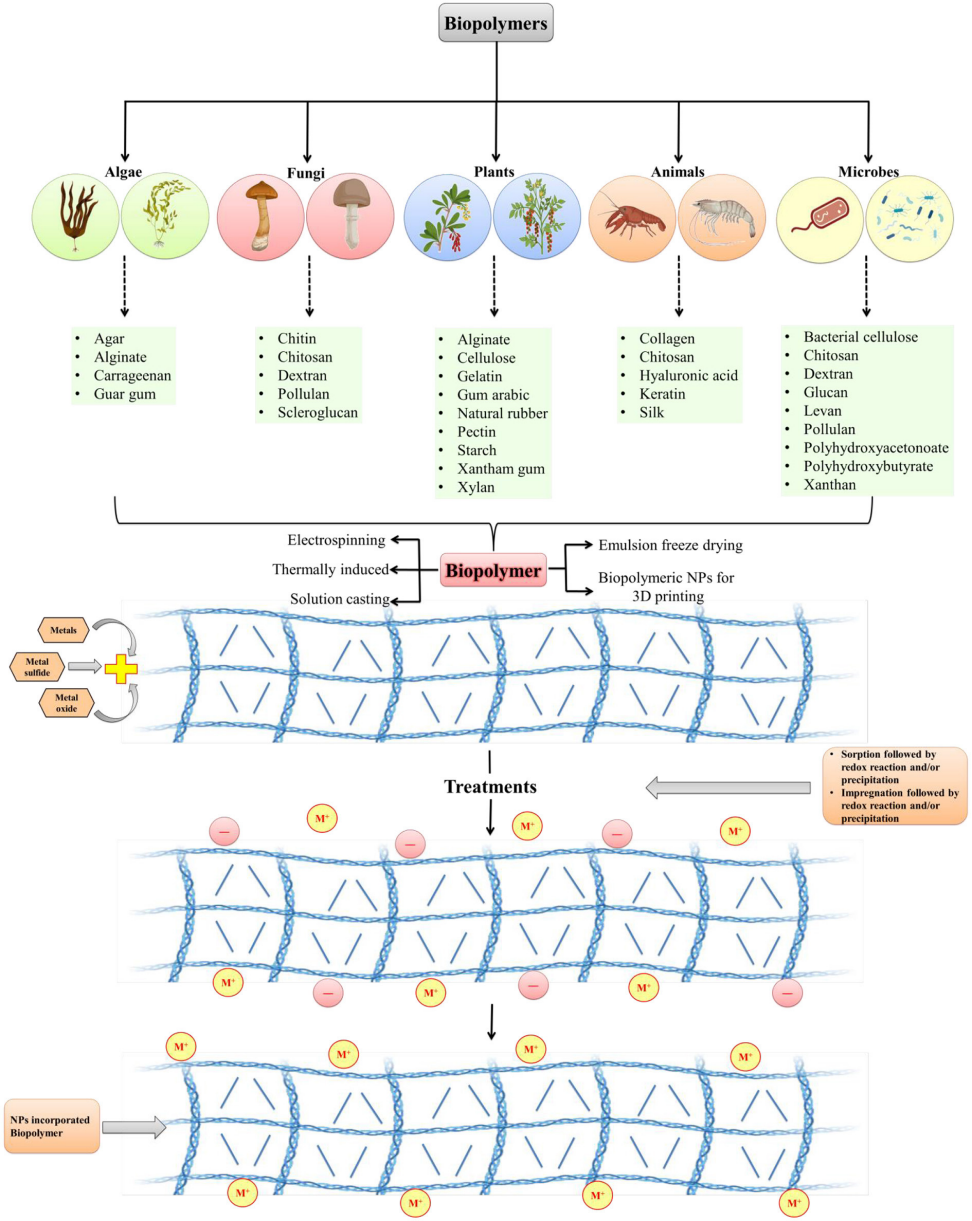
Development of biopolymeric nanoparticles from various sources (modified and adapted from Sarkar et al. (57).

Biopolymer composites integrate the necessary qualities of two or more acceptable materials to improve the physiological and mechanical needs of biomedical regions. Because of their long-term stability, chemical substances utilized in standard processes to produce polymeric nanomaterials may cause environmental impact. Biopolymers, on the other hand, are frequently created from non-toxic monomers and are carbon-neutral. It is easier to make nanomaterials from biopolymers since they are deposited at the nanoscale. Biopolymers have been found in plants, algae, fungi, bacteria, and animals. Macromolecules include starch, alginate, chitosan, dextran, and chitin (Figure 1). There are several compounds like pectin, cellulose, lignin, and hyaluronic acid, that are used to create nanomaterials composites. In the medical field, such as in the treatment of cancer, nano biocomposites have shown encouraging outcomes in recent years such as biodegradable substances, and chitosan nanoparticles are employed to deliver anticancer drugs (58). Biopolymers are made up of diverse components and have varied physiological properties. Biopolymeric nanomaterials can be made by attaching metals to biopolymers. To construct molecular capsules, biopolymers, in particular, utilize intramolecular hydrogen bonding. Starch, for example, can be used to create polymeric nanocomposites by combining metals or metal oxides. Chitosan is also used in nanotechnology applications because of its extensive compatibility (59). To make nanomaterials, silver (Ag) can be supramolecularly incorporated into starch (60). Nanomaterials can be incorporated into biopolymers by impregnation or absorption. Physical features of NPs can differ from macro-sized bulk materials due to their greater surface area and reactivity. Nanocapsules or nanospheres can be manufactured depending on the manufacturing approach (61). Spectroscopy, microscopy, and others are among the techniques used for NPs characterization.

Biopolymers and their derivatives are widely utilized, and studied in the creation of hydrogels. Chemical and physical methods of producing biomaterial-based hydrogels can be distinguished. The chemical process of incorporating substituent groups into biopolymer structures, strengthening and developing new functional bioactivities as a result. Covalent bonds between the utilized polymers are created via free radical polymerization, graft copolymerization, and other polymerization techniques (6, 62, 63). The chemical reaction within the hydrogel creates everlasting bonds in the polymer system. However, reversible physical interactions such as van der Waals attraction, hydrogen bonding, polymer chain entanglements, ionic interactions, and crystallite associations hold the hydrogels together during the physical process (64). There are several nanomaterials based biopolymers employed for effective delivery of bioactives for cancer therapy (Table 2).

**Table 2:** Nanoparticles based numerous biopolymeric compounds and their action in various cancer treatments.

**S. No.**	**Biopolymeric nanoparticles**	**Size (nm)**	**Polymer component**	**Actions**	**Bioactive molecules**	**Effects**	**Applications**	**References**
1.	Andrographolide analog chitosan based NPs	112–240	Octyl-grafted succinyl chitosan, naphthyl-grafted succinyl chitosan, and benzyl-grafted succinyl chitosan	Delivering anticancer medications to the sites of colon cancer	Andrographolide analog	Induces apoptosis	Colon cancer	(65)
2.	DOX-verapamil/MPEG-PLA NPs	25	MPEG-PLA	Co-delivery system –efficiently chemotherapeutic agents and coencapsulate verapamil	Doxorubicin, Verapamil	Tumor suppression	Ovarian cancer	(66)
3.	Linoleic acid conjugated SN38-loaded NPs	109	PEO-PBO diblock copolymer	–	Linoleic acid conjugated SN38	EBNPs inhibit growth and promote uptake in cancer cells	Colorectal cancer	(67)
4.	Curcumin- loaded Polymeric poly NPs	200	PLGA	Increased serum stability over free curcumin	Curcumin	Irradiating tumor cells at a low dose produces cytotoxic effects that inhibit tumor growth	Ovarian cancer	(68)
5.	Epidermal growth factor receptor-targeted lipid polymeric NPs	141.6	EGF-PEG-DSPE	Targeting the drug delivery	Cisplatin and Doxorubicin	Enhanced cytotoxicity with anticancer activity	Lung cancer	(69)
6.	Chondroitin sulfate functionalized campththecin-loaded polymeric NPs	289	Chitosan	Targeted drug delivery	Campththecin	Promote apoptosis	Colon cancer	(70)
7.	Albendazole-loaded polyurethane NPs	128.1	Polyurethane	Better medication delivery	Albendazole	Increase the anticancer efficacy *via* inducing apoptosis	Breast cancer	(71)
8.	Bortezomib loaded polymeric NPs	199.7	HPLA-BT	Higher drug load and its delivery	Bortezomib	Significant anticancer activity and shows higher cytotoxic effects	Breast cancer	(72)
9.	Gemcitabine NPs conjugated with linoleic acid.	~ 150	Linoleic acid	High drug-load, improved intracellular uptake and controlled release	Gemcitabine	Induce apoptosis and improve cytotoxic activity	Thyroid cancer	(73)
11.	Platinum–curcumin complexes loaded into pH and redox dual-responsive NPs	~ 100	mPEG-SS-PBAE-PLGA	Synergistic anticancer effects and control intracellular release	Platinum–curcumin	Improved anti-metastatic activity and synergistic anticancer implications	Lung cancer	(74)
12.	Uncariatomentosa extract -PLGA & UTPCL	300	PCL and PLGA	Better drug delivery	Uncariatomentosa extract	–	Prostate cancer	(75)

*DOX, Doxorubicin; PBA, Phenylboronic acid; PLA, Polylactic Acid; PLGA, Polylactide-co-glycolide; HPLA-BT, N-(2- hydroxypropyl) methacrylamide (HPMA)-PLA-Biotin; MPEG-PLA, Methoxy poly(ethylene glycol)- poly(lactide) copolymer; PEO-PBO, poly (ethylene oxide)-poly (butylene oxide) DSPE-PEG, 1,2-Distearoyl-sn-Glycero-3-Phosphoethanolamine- polyethylene glycol; PBAE, Poly-beta-amino ester*.

## Targeted delivery of bioactives *via* biopolymeric nanoparticles for cancer therapy

**Figure 2:**
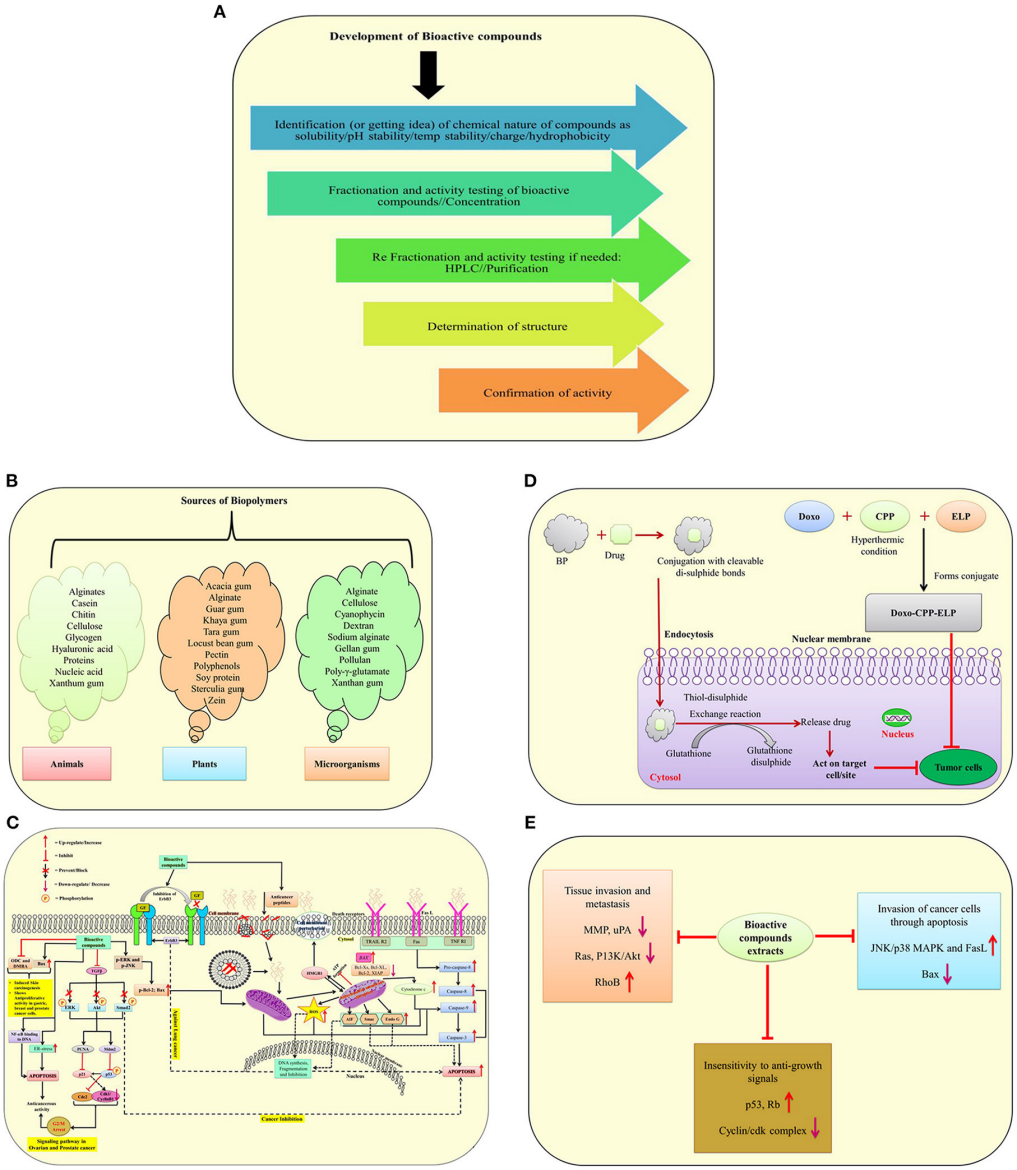
A schematic illustration of bioactive molecule sources and development, as well as their involvement in drug delivery systems and molecular mechanisms against different cancer cells. **(A)** Development of bioactive compounds; **(B)** Sources of biopolymers from plants, animals and microorganisms; **(C)** Mode of action of anticancerous molecules via TGF–β signaling pathway, death receptor, mitochondrial receptor–induced pathways against cancer cells; **(D)** A mechanism of suppressing tumor cells using biopolymers in conjunction with drugs; **(E)** The effect of bioactive molecules from plant extracts on the underlying mechanisms of cancer development [modified and adapted from Lin et al. (76)]. *ODC*, Ornithine decarboxylase; *DMBA*, 7,12-Dimethylbenz(α)anthracene; *Bcl-2*, B-cell lymphoma 2; *Bax*, Bcl-2-associated X protein; *NF-Kb*, Transcription factor; *p-ERK*, Protein kinase RNA-like ER kinase; *ROS*, Reactive oxygen species; *ENK*, c-Jun-N terminal kinase; *AIF*, Apoptosis-inducing factor; *FasL*, Fas Ligand; *ErbB3*, Receptor tyrosine kinase; *TGF-*β, Transforming growth factor beta; *ERK*, Extracellular-signal-regulated kinase; *AKT1*, Serine/threonine-protein kinase; *SMAD2*, SMAD family member 2; *Mdm2*, Mouse double minute 2 homolog; *PCNA*, Proliferating cell nuclear antigen; *p53 and p21*, Tumor protein; *WAF1/CIP1*, Wildtype activating factor-1/cyclin-dependent kinase inhibitory protein-1; *cdc2*, Cell division cycle 2; *HMGB1*, High mobility group box 1; *Smac, Second* mitochondria-derived activator of caspase; *EndoG*, Endonuclease G; *ER*, Endoplasmic reticulum; *TRAIL*, Tumor necrosis factor (TNF)-related apoptosis-inducing ligand; *TNF RI*, Tumor necrosis factor receptor 1; *Rb*, Retinoblastoma protein; *PI3K*, Phosphatidylinositol-3-kinase; *uPA*, Urokinase plasminogen activator; *BP*, Biopolymers.

According to the World Health Organization, cancer is one of the leading causes of mortality, with more than twenty million people expected to die by the year 2025 (1). Chemotherapeutic medicines work by interfering with DNA synthesis in rapidly dividing cancer cells, slowed DNA replication, reducing therapeutic efficacy and generating a significant mortality rate in cancer patients due to their large doses (4). Bioactive molecule sources and development, as well as their involvement in drug delivery systems and molecular mechanisms against different cancer cells, are illustrated in (Figures 2A–E). Natural bioactive chemicals have an impact on cellular metabolism and signaling pathways, and they can be used to cure cancer (Figure 2C). Natural bioactive chemicals have a favorable effect on cancer treatment, according to results from both *in vivo* and *in vitro* trials (10). A study reported in cancer reports showed that resveratrol NPs has enhanced anticancer activity both *in vitro* and *in vivo* (77). Biopolymers as NPs are increasingly being employed as a different way to deliver bioactive compounds/drugs or biological macromolecules to specific body regions. Polymer nanoparticles are a novel way of medication delivery. As a result, the primary goal of biopolymer-based nanomedicine is to deliver therapeutics just to cancer cells, increasing efficacy while reducing toxicity. Nanoparticles containing natural bioactives for targeted medication delivery in various tissues enhanced bioavailability, reduced side effects, improved *in vivo* stability, and improved target– specific activity of bioactive chemicals in cancer treatment. In the year 2020, Mousa et al. (78) reported that nanoformulation of 3, 3′-diindolylmethane (DIM) and ellagic acid showed more effective suppression of cell viability in pancreatic cancer, tumor weight, and tumor angiogenesis as compared to with these bioactive compounds alone. New formulation strategies on lipid–based delivery systems as nanocarriers also showed improved bio–efficacy under *in vivo* conditions (79).

Biopolymer-based nanocarriers, liposomes, dendrimers, lipid-based NPs, polymeric micelles, and other new drug delivery technologies have been discovered in the last two decades that allow bioactive chemicals to be delivered into the site of action (80). Proteins (albumin, gelatin, and milk proteins), polysaccharides (cellulose, starch, pectin, chitosan, dextran and sodium alginate), and nucleic acids are examples of biopolymers (81). Biopolymer NPs were found to be effective in delivering bioactive chemicals to malignant cells in both *in vivo* and *in vitro* investigations. Biopolymeric nanoparticles containing bioactive substances have several advantages over other current delivery technologies, including non water–soluble drug delivery, stability protection, and targeted medication delivery to specific tumor cells (82). Nanoparticles deliver the therapeutic chemical, which could be natural bioactive or synthetic, directly into the target tissue/organ. Nanoparticles have a lot of potential as molecular transporters since they can deliver treatments to malignant cells without harming healthy cells (83). Polymeric pharmaceuticals, polymeric micelles, polyplexes, polymer–drug conjugates, and polymer–protein conjugates are all examples of biopolymers utilized in therapy (84).

The ways NPs are administered, as well as their physicochemical properties, have a big impact on therapeutic efficacy and biological consequences (Figure 2C). Every mode of administration has advantages and disadvantages, but the oral route of delivery is the safest and most convenient way to treat cancer. The oral mode of administration, on the other hand, has drawbacks such as gastrointestinal degradation, insolubility, and reduced absorption. As a result, current anticancer compositions aren't suited for use as oral chemotherapy. The use of biopolymers for oral delivery platforms has the potential to overcome some of the problems associated with the delivery of chemotherapy drugs. Biopolymeric formulations prevent NPs from pH degradation, enhance water solubility and stability, and target particular binding sites for selective absorption and drug release (Figure 2D) (85). Future formulations from diverse plants with anticancer properties could significantly boost the potency of anticancer medications for cancer therapy (9). The potency of recognized naturally occurring bioactive chemicals has been increased through the use of nanomaterials. In the future, herbal compounds might play a bigger role in the delivery mechanism for nano biocomposites mediated therapeutic molecules. Further research is required in this area.

## Challenges and future perspectives

Every day, there are large number of patients who have advance tumors and most common concern regarding the therapy is the effectiveness and the expense of cancer medication. The effectiveness and cost of pharmaceuticals have not altered dramatically as a result of the development of new technology and drugs. Not only is the efficacy of new treatments a source of concern in India; but wealthier countries such as the United States and Europe are also experiencing issues with cancer drug delivery systems. Nanotechnology is one of the most exciting fields in the area of drug delivery systems to treat non–curable cancer disorders, especially in such a crucial situation. Nanoparticles are utilized to target and deliver drugs to cancer/tumor cells while preserving the physiology of non-tumorous cells. Researchers are working to make more consistent NPs so that the right amount of harmless therapeutics can be delivered to the right place. Metal–based (gold and silver) NPs production has progressed significantly for their applications in diagnostic and therapeutic purposes. The understanding of treatment mechanism at the molecular level will expand bioactive compounds applications for cancer therapy. Because of the regulated release of specific medications at targeted places, assessment of events during drug interaction at the tissue/cellular level, and prediction has not yet been found completely, and its application in cancer therapy/diagnosis is still limited. Animal research will provide important information on the possible use of nanoformulations as medicinal therapeutics. Acute and chronic studies are required to assess the possible harm of any type of anticancerous therapeutic molecule to humans and the environment, and their affordability is another area of research that requires further input.

## Conclusions

Drugs have numerous obstacles, thus researchers are focusing on herbal medicine to reduce drug side effects and improve their effectiveness through the use of appropriate effective delivery systems. Preclinical and *in vitro* investigations have revealed that NPs are particularly effective at delivering chemotherapeutic medicines, molecules as well as natural components to specific tumor sites. Nanoparticles have significant advantages over alternative drug delivery systems in terms of high specificity, high stability, controlled drug release, and high drug–carrying capacity, and the ability to transport both hydrophobic and hydrophilic drug molecules. Because of their controlled release, subcellular size, and biocompatibility with cells, polymeric NPs are now widely used as drug/anticancer bioactives delivery systems in the pharmaceutical and medical areas for cancer therapy.

## Author contributions

NP: conceptualization. PS, PKS, and AG: writing—original draft, reviewing and editing. SS, RS, AG, RM, and VM: reviewing, editing, and drawing figures and tables. MT and NP: reviewing, editing and finalize the manuscript. All authors have read and agreed to the published version of the manuscript.

## Conflict of interest

The authors declare that the research was conducted in the absence of any commercial or financial relationships that could be construed as a potential conflict of interest.

## Publisher's note

All claims expressed in this article are solely those of the authors and do not necessarily represent those of their affiliated organizations, or those of the publisher, the editors and the reviewers. Any product that may be evaluated in this article, or claim that may be made by its manufacturer, is not guaranteed or endorsed by the publisher.
